# Review: Receptor Targeted Nuclear Imaging of Breast Cancer

**DOI:** 10.3390/ijms18020260

**Published:** 2017-01-26

**Authors:** Simone U. Dalm, John Fred Verzijlbergen, Marion De Jong

**Affiliations:** Department of Radiology & Nuclear medicine, Erasmus MC, Wytemaweg 80, 3015CN Rotterdam, The Netherlands; j.verzijlbergen@erasmusmc.nl (J.F.V.); m.hendriks-dejong@erasmusmc.nl (M.D.J.)

**Keywords:** breast cancer, receptor targeted nuclear imaging, SPECT, PET, GRPR, SSTR, ER, PR, HER2

## Abstract

Receptor targeted nuclear imaging directed against molecular markers overexpressed on breast cancer (BC) cells offers a sensitive and specific method for BC imaging. Currently, a few targets such as estrogen receptor (ER), progesterone receptor (PR), human epidermal growth factor receptor 2 (HER2), somatostatin receptor (SSTR), and the gastrin releasing peptide receptor (GRPR) are being investigated for this purpose. Expression of these targets is BC subtype dependent and information that can be gained from lesion visualization is dependent on the target; ER-targeting radiotracers, e.g., can be used to monitor response to anti-estrogen treatment. Here we give an overview of the studies currently under investigation for receptor targeted nuclear imaging of BC. Main findings of imaging studies are summarized and (potential) purposes of lesion visualization by targeting these molecular markers are discussed. Since BC is a very heterogeneous disease and molecular target expression can vary per subtype, but also during disease progression or under influence of treatment, radiotracers for selected imaging purposes should be chosen carefully.

## 1. Introduction

Breast cancer (BC) is the most common cancer in women worldwide. In 2012, 167 million new BC cases were diagnosed and 522,000 people died of the disease [1]. BC is highly heterogenic and comprises of multiple histological subtypes e.g., luminal A, luminal B, human epidermal growth factor 2 (HER2)-driven, and basal-like tumors [2]. These histological subtypes are characterized by distinctive molecular patterns that play an important role in treatment and prognosis of the disease. The most important molecular tumor characteristics include estrogen receptor (ER), progesterone receptor (PR), and human epidermal growth factor receptor 2 (HER2) expression [2].

Our knowledge of BC has greatly expanded over the past years leading to new diagnostic and therapeutic methods, which positively influenced the mortality rate of the disease. The prognosis of metastatic BC is still poor, the estimated five-year survival being only 26% [3], and therefore new imaging and therapeutic methods are needed. Although BC is finally diagnosed by histology, imaging methods are indispensable for detection of the disease. Mammography is used for nationwide breast screening, in some cases supplemented with magnetic resonance imaging (MRI) or ultrasound. These methods are suited for screening purposes and detection of abnormal breast lesions but do not provide information on molecular characteristics such as biomarker expression. Imaging techniques that can provide such information can have added value, especially in highly heterogeneous cancer types. To fulfill this purpose, target-mediated nuclear imaging of BC is being investigated.

**Figure 1:**
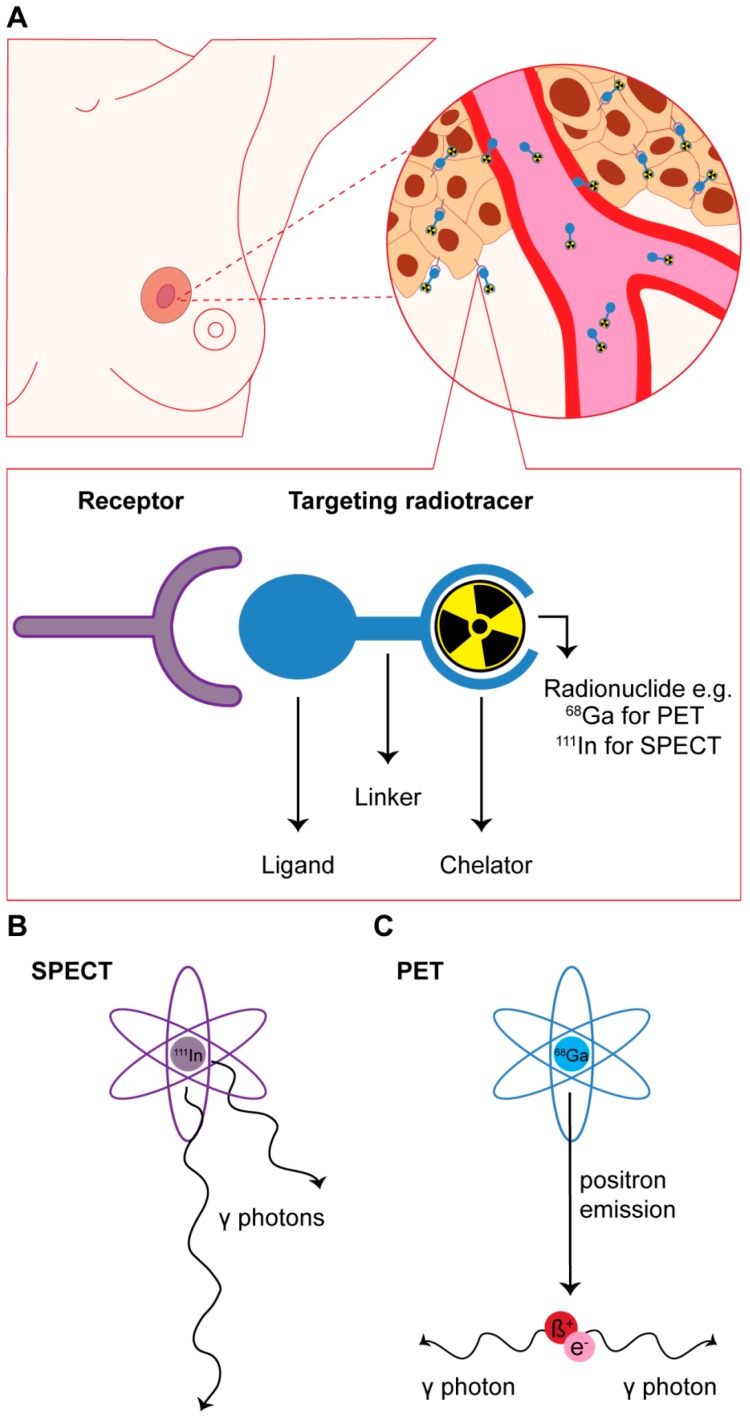
(**A**) Schematic overview of receptor targeted nuclear imaging. Ligands that can bind their targets overexpressed on breast cancer (BC) cells can be coupled to a chelator, often via a linker. The chelator enables labeling with radionuclides such as ^68^Ga and ^111^In that can be applied for imaging purposes; (**B**,**C**) drawing of the principles of radionuclides for single-photon emission computed tomography (SPECT) and positron emission tomography (PET) imaging. For SPECT imaging, γ-photons from radionuclides such as ^111^In are captured by detectors at multiple positions around the longitudinal axis of the patient. For PET imaging positrons emitted from a radionuclide such as ^68^Ga interact with electrons which results in the production of 2 γ-photons. These photons are picked up by opposing detectors installed in a ring-like pattern.

In nuclear medicine, such target-mediated imaging is successfully applied for imaging of, e.g., neuroendocrine tumors [4,5]. This approach uses the molecular expression pattern of tumors for targeting. Molecules (e.g., receptors, transporters, and enzymes) overexpressed on cancer cells can be targeted with synthesized target ligands (e.g., peptide analogs, antibodies, affibodies, and nanobodies) that bind to the target with high affinity and specificity (Figure 1A).

Depending on the radionuclide that these targeting agents are conjugated with, single-photon emission computed tomography (SPECT) or positron emission tomography (PET) can be performed. SPECT and PET are functional, highly sensitive nuclear imaging methods based on the detection of γ-photons directly or indirectly derived from γ-emitting (e.g., ^111^In) or positron-emitting (e.g., ^68^Ga) radionuclides, respectively (Figure 1B,C). Combining SPECT or PET with computed tomography (CT) or MRI provides functional imaging information in combination with high resolution imaging of anatomical structures [6,7]. In the review by Pattion et al. [8] and the paper by Ziegler et al. [9] the mechanisms of SPECT and PET imaging are described in more detail. With respect to BC imaging, dedicated SPECT and PET imaging devices have been developed that have a higher resolution and thus better diagnostic accuracy than whole body SPECT and PET systems [10,11].

Targeted nuclear imaging may potentially be used for disease characterization, disease visualization (e.g., preoperative scanning, disease staging by visualization of regional and distant metastases and/or detection of relapse) and in some cases to predict outcome of therapy and to monitor/evaluate treatment response. For screening purposes, however, the use of targeted nuclear imaging might be less suited since its success rate is dependent on sufficient target expression, and because of the relatively high costs and the radiation burden associated with this method.

In the past years a number of molecular targets for receptor targeted nuclear imaging of BC have been identified and are currently under investigation: hormone receptors, HER2, the somatostatin receptor (SSTR), the gastrin releasing peptide receptor (GRPR), folate receptor (FR), C-X-C chemokine receptor type 4 (CXCR4), neuropeptide Y receptor Y1 (NPY1R), and vasoactive intestinal polypeptide receptor 1 (VIP-R1). In this review we describe these targets and discuss ongoing investigations and the prospects of BC receptor targeted nuclear imaging.

This review focuses on the molecular targets mentioned above. Other radiotracers under investigation for nuclear imaging of BC including radiotracers that accumulate in cells due to (over)expression of functional transporters or higher metabolism in BC cells (e.g., ^18^F-fluorodeoxyglucose (FDG)) are beyond the scope of this review.

## 2. Targeting of Hormone Receptors for Nuclear Imaging

**Figure 2:**
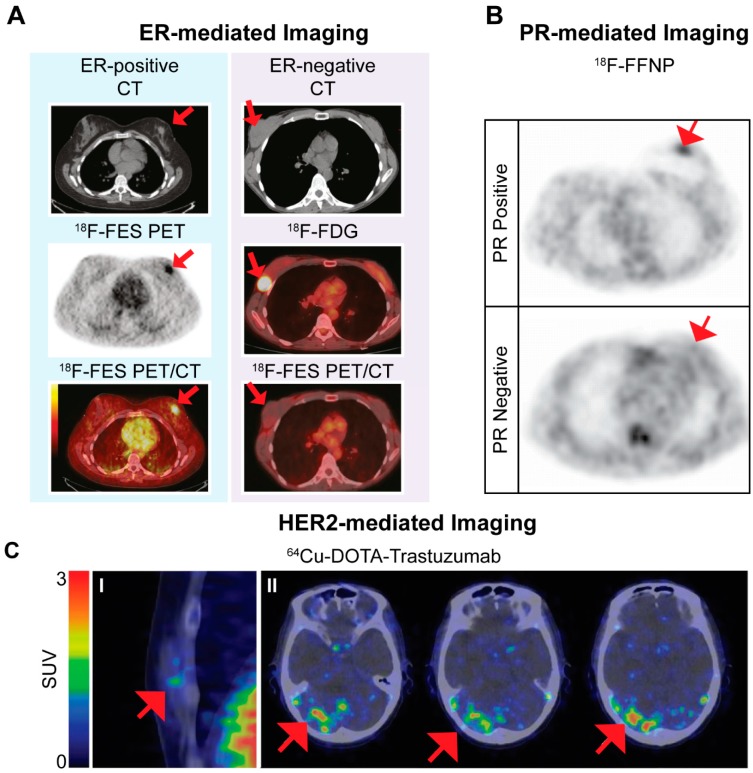
Examples of images using the estrogen receptor (ER)-targeted radiotracer ^18^F-FES (**A**); progesterone receptor (PR)-targeted radiotracer ^18^F-FFNP (**B**) and the human epidermal growth factor receptor 2 (HER2)-targeted radiotracer ^64^Cu-DOTA-trastuzumab obtained in breast cancer (BC) patients. Images are adapted from Gemignani et al. [17], Dehdashti et al. [27] and Tamura et al. [31], respectively. (**A**) ^18^F-FES PET images of an ER-positive and an ER-negative BC lesion. The ER-positive BC lesion visualized by ^18^F-FES PET corresponds with a 2–3 cm lesion seen on CT and is confirmed on the ^18^F-FES PET/CT images. The ER-negative BC lesion is visualized on CT and by ^18^F-FDG PET but shows no ^18^F-FES uptake. This research was originally published in *JNM.* **Gemignani et al. Feasibility and predictability of perioperative PET and estrogen receptor ligand in patients with invasive BC. *J. Nucl. Med.* 2013, *54*, 1697–1702.** © by the Society of Nuclear Medicine and Molecular Imaging, Inc. (Ann Arbor, MI, USA); (**B**) ^18^F-FFNP PET images in a patient with PR-positive and PR-negative BC. This research was originally published in *JNM.* **Dehdashti et al. Assessment of progesterone receptors in breast carcinoma by PET with 21-18f-fluoro-16α,17α-[(r)-(1′-α-furylmethylidene)dioxy]-19-norpregn-4-ene-3,20-dione. *J. Nucl. Med.* 2012, *53*, 363–370.** © by the Society of Nuclear Medicine and Molecular Imaging, Inc. (**C**) Examples of HER2-targeted imaging. In part I ^64^Cu-DOTA-trastuzumab PET images of a HER2-positive primary BC is shown. Red areas show high tracer uptake in blood vessels. Part II shows images of HER2-positive metastatic brain lesions identified by ^64^Cu-DOTA-trastuzumab PET imaging. Significant tracer uptake values were found in areas corresponding to brain metastatic lesions detected on MRI. Images in part I and II are from different patients. This research was originally published in *JNM.* **Tamura et al. ^64^Cu-DOTA-trastuzumab PET imaging in patients with HER2-positive BC. *J. Nucl. Med.* 2013, *4*, 1869–1875.** © by the Society of Nuclear Medicine and Molecular Imaging, Inc. Red arrows indicate cancer lesions in the images. SUV: standardized uptake value.

The ER is not only interesting for therapeutic targeting options, but also for imaging. ^18^F-FES, a fluorinated estradiol [12], is the most extensively studied ER-targeting PET radioligand in clinical trials. Studies have focused on the potential of ER-mediated nuclear imaging for visualization of ER-positive primary and metastatic BC lesions as well as the ability of the radioligand to predict response to anti-estrogen treatment. Five clinical studies reported on sensitivity and specificity of the radiotracer for tumor visualization; 69%–100% and 80%–100%, respectively [13,14,15,16,17]. Furthermore, ^18^F-FES imaging was used to predict response to anti-estrogen treatment prior to and in early phases of therapy. High uptake of ^18^F-FES prior to treatment indicates the presence of ER, which is necessary for a positive therapy response, while a decrease of ^18^F-FES uptake in early phases of treatment is an indication of successful treatment. Figure 2A shows an example of the use of ^18^F-FES for predicting therapy response in BC patients. Up to now, positive and negative predictive values of 65% and 88%, respectively, were reported for pre-therapy scanning in relation to anti-estrogen treatment [18,19,20,21]. Since ^18^F-FDG (which reflects glucose metabolism) is the most widely used PET tracer for evaluation of treatment response [22,23], He et al. [24] compared the use of ^18^F-FES and ^18^F-FDG in a preclinical setting and reported that ^18^F-FES PET/CT was superior to ^18^F-FDG for predicting response to endocrine therapy. Following the above-mentioned positive results, a substantial number of clinical trials using ^18^F-FES for BC imaging have started and are still ongoing.

Based on current findings, ^18^F-FES could be useful for disease characterization by determining ER expression of BC lesions (offering a less invasive method than immunostaining on biopsy material), disease staging, and the use of the radiotracer to predict and monitor therapy response.

Because expression of the PR is an estrogen-regulated process, the primary focus was on the development of ER-targeted radiotracers. However, ER-targeting radiotracers are not always efficient in patients treated with anti-estrogens since these molecules bind to the ER as well, rendering the receptor unavailable for radiotracer binding, such as interim monitoring of treatment efficacy. In this case, PR-targeted radiotracers might be useful. Furthermore, similar to ER status, PR-targeting radiotracers offer a less invasive method for determining PR status of breast lesions. A number of PR-targeting radiotracers have been synthesized and investigated in preclinical and clinical studies [25,26]. The most successful PR-targeted radiotracer, ^18^F-FFNP, was used in a clinical pilot study successfully identifying 15/16 PR-positive BCs [27]. Figure 2B shows an example of ^18^F-FFNP PET images in a PR-positive and a PR-negative BC patient. Previous research reported a decrease in PR expression after successful anti-estrogen treatment as a result of inhibition of ER-activated pathways [28] and preclinical studies investigating the potential of ^18^F-FFNP PET imaging to predict response to anti-estrogen treatment have been performed with promising results [29,30].

To date, clinical data on PR-targeted nuclear imaging is limited, but the reported findings indicate that the potential application of PR radioligands lies in disease characterization by determination of PR expression and therapy assessment after endocrine treatment.

## 3. Human Epidermal Growth Factor Receptor 2 (HER2)-Targeted Imaging

Similar to hormone receptors, HER2 expression in BC is not only of interest for therapeutic interventions but also for imaging. HER2-targeted nuclear imaging has been tested in preclinical and clinical studies using both radiolabeled monoclonal antibodies, radiolabeled affibodies, radiolabeled nanobodies, and radiolabeled antibody fragments. Monoclonal antibodies used for therapy of HER2-expressing BCs were radiolabeled with different radionuclides enabling both SPECT and PET imaging. Following positive results from preclinical studies, radiolabeled trastuzumab was investigated in clinical studies for its ability to visualize HER2-positive BC lesions [31,32,33,34,35,36,37]. Figure 2C shows an example of PET images acquired after injecting HER2-positive BC patients with radiolabeled trastuzumab. The main purpose of studying HER2-targeted nuclear imaging was to predict response to treatment with trastuzumab as well as other types of treatment, and to predict trastuzumab-related toxicity. The results of clinical studies were variable, limiting factors being poor visualization of liver metastases due to high background uptake in the liver and suboptimal imaging of HER2-positive lesions if no unlabeled trastuzumab was pre-administered. Remarkably, two studies demonstrated that ^64^Cu-Trastuzumab was able to detect HER2-positive breast lesions that could not be detected by ^18^F-FDG PET [31,34]. Furthermore, treatment of HER2-positive BC patients with a combination of trastuzumab and paclitaxel or a heat shock protein 90 inhibitor NVP-AUY922 led to a decrease in uptake of radiolabeled trastuzumab, indicating that the radiotracer can be used to assess response to these types of treatment [35,36]. In a recent study by Gebhart et al. [38] the ability of ^89^Zr-trastuzumab imaging to predict response to treatment with trastuzumab emtasine was evaluated in 56 patients. The authors reported that HER2-targeted imaging combined with early metabolic response assessment by ^18^F-FDG PET predicted response to trastuzumab emtasine treatment and discriminated between patients that will or will not benefit from this type of therapy.

In contrast to antibodies, the smaller affibody molecules have relatively fast uptake and clearance rates, resulting in a lower radiation burden for patients and offering the opportunity to scan patients at earlier time points after administration of the radiotracer. Two clinical studies have been performed evaluating the use of radiolabeled HER2-targeting affibodies in patients, which resulted in successful imaging of HER2-positive BC lesions [39,40]. However, similar to the results with radiolabeled trastuzumab, imaging of liver metastases was difficult because of high physiological uptake in the liver. Additionally, radiolabeled HER2-targeting nanobodies that can be labeled with different radionuclides (e.g., ^18^F, ^68^Ga, and ^99m^Tc) were synthesized and applied for HER2 visualization [41,42,43]. The majority of these nanobodies are still under investigation in a preclinical setting, but a recent clinical study by Keyaerts and Xavier et al. [44] reported on the use of ^68^Ga-HER2-Nanobody in BC patients. Although not the primary goal of the study, both primary and metastatic BC lesions were successfully visualized. Furthermore, biodistribution was favorable and no toxicity was reported. In addition, radiolabeled HER2 antibody fragments were synthesized and tested preclinically as well as clinically. Although preclinical studies were successful [45,46,47,48,49], only a few clinical studies have been reported and results were disappointing since radiotracer uptake in tumors was absent or low [50,51]. Also, radiolabeled HER2-targeting RNA aptamers were synthesized for targeting HER2-positive BC lesions [52]. These studies are still in a preclinical setting and their advantage to HER2-targeting antibodies, affibodies, and nanobodies remains to be established.

Current findings indicate that HER2-targeted imaging could be applied for disease characterization by determination of HER2 expression of breast tumors, disease staging and to monitor therapy responses.

## 4. Somatostatin Receptor (SSTR)-Mediated BC Imaging

**Figure 3:**
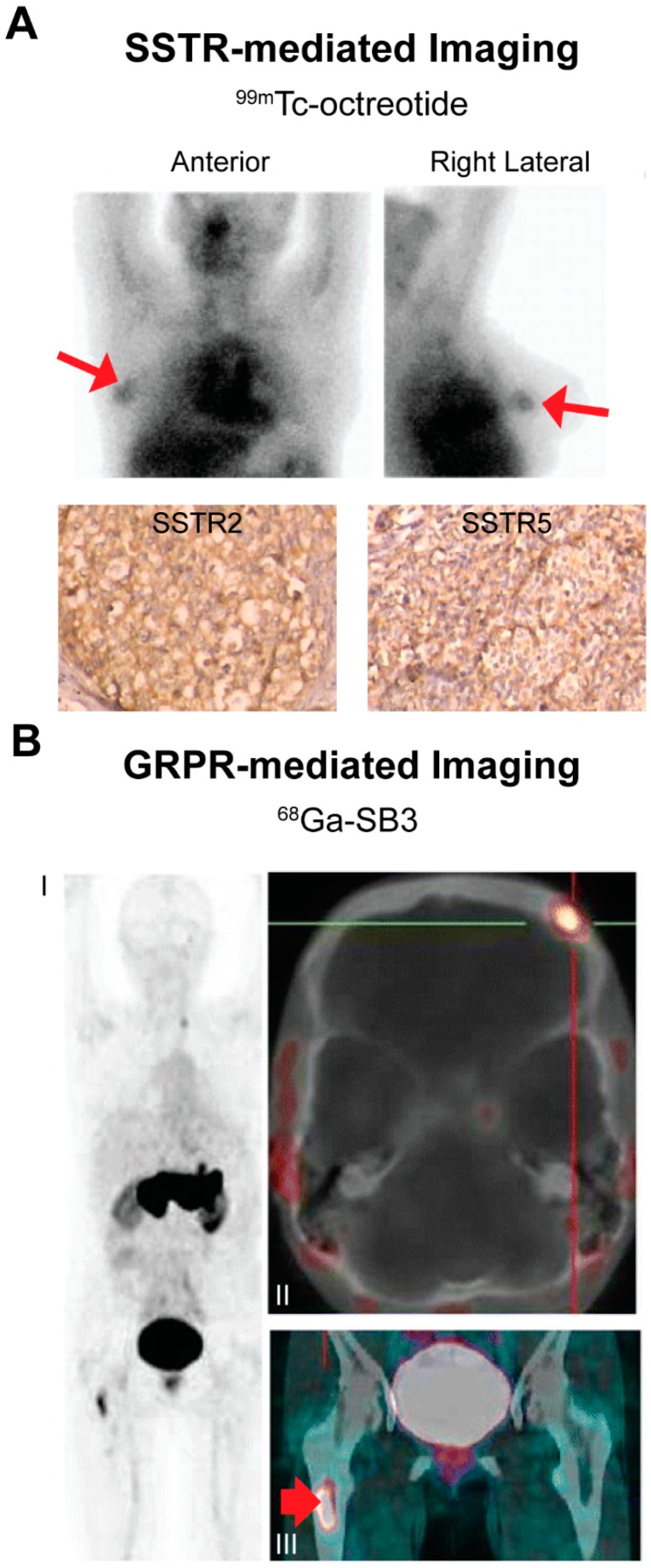
Examples of somatostatin receptor (SSTR)- (**A**) and gastrin releasing peptide receptor (GRPR)- (**B**) mediated breast cancer (BC) imaging. Images are adapted from studies by Wang et al. [64] and Maina and Bergsma et al. [80], respectively. (**A**) ^99m^Tc-octreotide (a SSTR-targeting radiotracer) scintigraphy identifying SSTR-positive BC tumors. SSTR2 and SSTR5 expression on cancer cells was demonstrated by immunohistochemistry stainings. This research was reprinted from **Wang et al. The role of technetium-99m-labeled octreotide acetate scintigraphy in suspected breast cancer and correlates with expression of SSTR. *Nucl. Med. Biol.* 2008, *35*, 665–671.** Copyright, with permission from Elsevier [64]; (**B**) ^68^Ga-SB3 scan in a BC patient, demonstrating GRPR-positive bone metastasis in the skull: frontal bone on the left side (part II, SUV_max_ 2.4) and bone marrow metastasis in the right proximal femur (part III, arrow, SUV_max_ 7.8). Part I MIP; part II fused axial PET/CT image and part III fused coronal PET/CT image. SUV_max_: maximum standardized uptake value. This research was originally published in **Maina and Bergsma et al. Preclinical and first clinical experience with the gastrin-releasing peptide receptor-antagonist [^68^Ga]SB3 and PET/CT. *Eur. J. Nucl. Med. Mol. Imaging* 2015, *43*, 964–973** [80]; © Springer-Velag Berlin Heidelberg 2015, with permission of Springer.

Receptor-mediated nuclear imaging is successfully used in neuroendocrine tumor patients by targeting SSTRs overexpressed on neuroendocrine tumor cells using SSTR-binding radioligands. Next to neuroendocrine tumors, SSTR expression has also been reported on BC cells [53,54]. Since radiolabeled peptide analogs targeting these receptors were available, several preclinical and clinical studies have been performed targeting these receptors for imaging purposes. In the preclinical study by Chereau et al. [55], ^68^Ga-DOTA-TOC imaging was compared to ^18^F-FDG PET in a BC xenograft mouse model resulting in two times higher uptake of ^68^Ga-DOTA-TOC compared to ^18^F-FDG. Concerning clinical investigations, in our previous review we discussed earlier clinical studies [56,57,58,59,60,61,62,63,64,65,66] on SSTR-mediated imaging, showing very variable sensitivities and specificities ranging from 36%–100% and 22%–100%, respectively [67]. Figure 3A shows an example of SSTR-mediated imaging in BC patients. Limiting factors for successful BC targeting were low and heterogeneous SSTR expression, appropriate patient selection, the use of radiolabeled peptide analogs with suboptimal receptor affinity and imaging equipment with low spatial resolution. For successful receptor-mediated imaging the expression of the target should be sufficiently high. Since low and heterogeneous SSTR expression was reported as a limiting factor for successful BC imaging, the question is whether SSTR is a suitable target for receptor targeted nuclear BC imaging. However, another limiting factor of the previous studies was non-appropriate patient selection. Since BC is a very heterogeneous disease, SSTR expression between BC subtypes may vary. We and others showed higher SSTR expression in ER-positive BC compared to ER-negative BC, identifying ER-positive BC subtypes as the most suitable subtypes for SSTR-mediated imaging [68,69,70,71]. If we only focus on these BC subtypes, which account for the majority of the breast tumors, SSTR-mediated BC imaging might be more successful. Furthermore, we studied *SSTR* mRNA expression of primary BCs vs. *SSTR* mRNA expression of regional and distant metastases and demonstrated that there was no significant difference in *SSTR* mRNA expression levels of primary tumors and corresponding regional lymph node metastases as well as lung and brain metastases [72]. Previous studies have been performed with radiolabeled octreotide, which has a lower SSTR affinity compared to the currently used radiolabeled somatostatin analogs, including Tyr^3^-octreotate [73]. In addition, lower spatial resolution planar imaging was used in earlier studies in comparison to currently available whole body and dedicated SPECT and PET techniques [6,10,11]. Another noteworthy recent development is the application of SSTR antagonists that have shown to be superior to SSTR agonists for neuroendocrine tumor targeting [74,75,76,77,78]. This enhanced tumor targeting of SSTR antagonists was explained by the ability of receptor antagonists to bind more binding sites/receptors than receptor agonists [78]. Since SSTR expression in BC was reported to be low and heterogeneous, the use of antagonists is promising in this respect. Cescato et al. [77] reported 11 ± 4 times higher binding of an SSTR antagonist, ^177^Lu-DOTA-BASS, vs. the clinically used SSTR agonist ^177^Lu-DOTA-Tyr^3^-octreotate in seven human BC specimens. We recently reported on enhanced binding of the radiolabeled SSTR antagonist DOTA-JR11 vs. the radiolabeled SSTR agonist DOTA-Tyr^3^-octreotate in 40 BC specimens as well as superior imaging of a patient-derived BC xenograft mouse model post injection of the radiolabeled receptor agonist vs. the antagonist [79].

Thus, previous studies on SSTR-mediated imaging in BC performed under suboptimal conditions were not convincing, but with recent improvements as mentioned above, the outcome might change. Additional studies that benefit from these recent developments are needed to investigate the true potential of SSTR-mediated BC imaging. Based on previous studies, we speculate that the potential for SSTR-mediated imaging lies in disease staging and disease monitoring.

## 5. Gastrin Releasing Peptide Receptor (GRPR)-Mediated BC Imaging

The GRPR is a G-protein coupled receptor that is overexpressed on a high percentage of BCs. According to literature, 62%–96% of primary BCs express GRPR [81,82,83,84]. Over the past years, multiple GRPR-targeting radioligands have been described to target GRPR-expressing cancers. Although the majority of these were studied in prostate cancer, these studies expanded our knowledge on preferential radioligand properties and uptake in other/background organs. One example is the preference for radiolabeled GRPR antagonist instead of agonists for tumor targeting, since it is similar to what was observed for SSTR radioligands, superior binding of GRPR antagonists vs. agonists was reported [85]. Several preclinical studies have been performed demonstrating successful GRPR-mediated nuclear imaging using SPECT and PET in BC mouse models [84,86,87]. In the study by Prignon et al. [86], GRPR-mediated imaging was compared to ^18^F-FDG PET for tumor visualization and disease monitoring after endocrine therapy, resulting in a significant decrease in uptake of the GRPR radiotracer, ^68^Ga-AMBA, in treated and non-treated animals while no significant difference in ^18^F-FDG uptake between the treated and non-treated group was observed. Also, we reported that high *GRPR* mRNA expression levels were associated with improved progression free survival after first line tamoxifen (Nolvadex) treatment, indicating that GRPR expression has predictive value for response to tamoxifen treatment [68]. In the same study, we reported on higher GRPR expression in ER-positive tumors, identifying specific BC patients suited for the application of radiotracers targeting this receptor. Furthermore, we recently reported that BC metastases from GRPR-positive primary BCs also express GRPR, indicating that this imaging method can be applied in both primary and metastatic disease [72]. Although results obtained from preclinical studies are promising, to date only a few clinical studies have been performed on GRPR-mediated nuclear BC imaging. In a study by Maina et al. [80], four out of eight breast tumors were successfully visualized in patients with advanced disease using ^68^Ga-SB3, a radiolabeled GRPR antagonist. Scan outcomes were not related to ER expression in this study. Stoykow et al. [88] showed successful imaging in 13 out of 18 patients with another ^68^Ga labeled GRPR-antagonist, ^68^Ga-RM2. Positive imaging results were correlated with ER expression in accordance with our findings [68], confirming the potential of GRPR-mediated imaging in ER-positive patients. A ^68^Ga-SB3 PET image from the study by Maina and Bergsma et al. [88] is presented in Figure 3B. Although more clinical studies on the application of GRPR radioligands for BC imaging are needed, current findings suggest that GRPR-targeted imaging might be used successfully for disease staging and therapy evaluation in ER-positive patients.

## 6. Other Targets

Next to the above-mentioned targets there are some other interesting targets that are not extensively studied in BC (yet). FR-targeting radiotracers have been applied for BC imaging. Overexpression of the FR was associated with basal-like BCs [89]. In a clinical study, successful SPECT imaging using a ^99m^Tc labeled folate tracer was performed in three out of six BC patients [90]. Radiotracers targeting folate receptors are currently in clinical trials mainly focusing on targeting of ovarian cancer.

High expression of CXCR4 and its association with invasive disease was reported in primary and metastatic BC cells [91]. Radiotracers targeting this receptor for imaging purposes were investigated in BC in a few preclinical studies and one clinical study [92,93,94]. The result of the clinical study using the CXCR4 radiotracer ^68^Ga-pentixafor was disappointing and ^18^F-FDG seems superior to ^68^Ga-pentixafor for BC imaging. However, only few BC patients were included in this study and larger clinical studies are needed to accurately determine the value of CXCR4-mediated BC imaging. Since *CXCR4* mRNA expression was associated with ER-negative tumors [68], successful targeting would offer new imaging opportunities for this patient group.

NPY1R expression has been reported on 85% of breast tumors and radiotracers for BC targeting have been synthesized [95,96,97,98]. Highest expression of the receptors was reported on triple negative BCs [99]. Up to date, proof of successful imaging using these radiotracers is very scarce, but the available data appear promising.

Furthermore, overexpression of VIP-R on BC cells was reported in multiple studies and radiotracers targeting these receptors were synthesized [100,101]. One preclinical and one clinical study described the use of radiolabeled VIP-R radiotracers (^18^F-dVIP and ^64^Cu-TP3805, respectively) for imaging purposes in BC patients [102,103]. In the clinical study by Thakur et al. [103] 20 out of 20 BCs were successfully imaged. The authors hypothesize that VIP-R type 1-mediated imaging can be used for early and accurate detection of BC because it is overexpressed on all BC cells in early phases of the disease. However, the high uptake of VIP-R type 1 targeted radiotracers in the lungs reported in studies performed in other cancer types should be kept in mind [104].

## 7. Future Outlooks

With the different targets discussed above being explored and available for receptor-mediated nuclear imaging of BC, several questions remain. How can receptor targeted nuclear imaging improve the care of BC patients and what is the best target for receptor targeted nuclear BC imaging?

**Figure 4:**
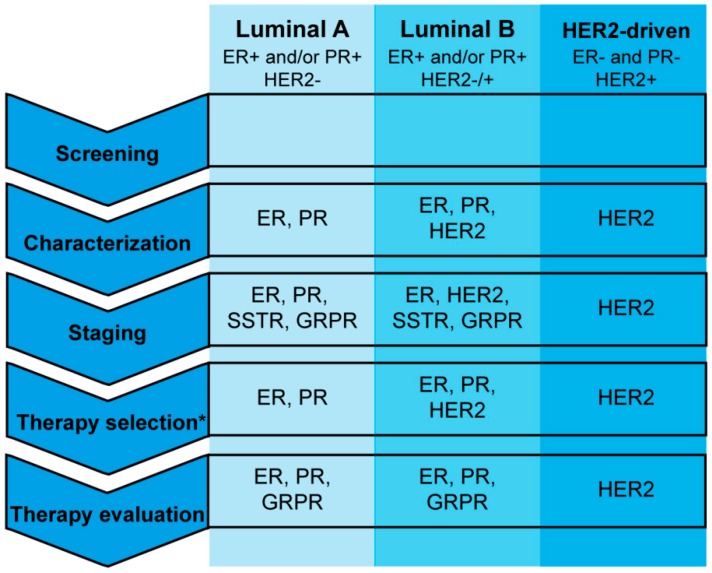
Overview of the (potential) clinical application of radiotracers targeting the estrogen receptor (ER), progesterone receptor (PR), human epidermal growth factor receptor 2 (HER2), somatostatin receptor (SSTR), and gastrin releasing peptide receptor (GRPR) for breast cancer imaging.* Therapy selection refers to currently clinically used hormonal and chemotherapies as discussed in this article.

**Figure 5:**
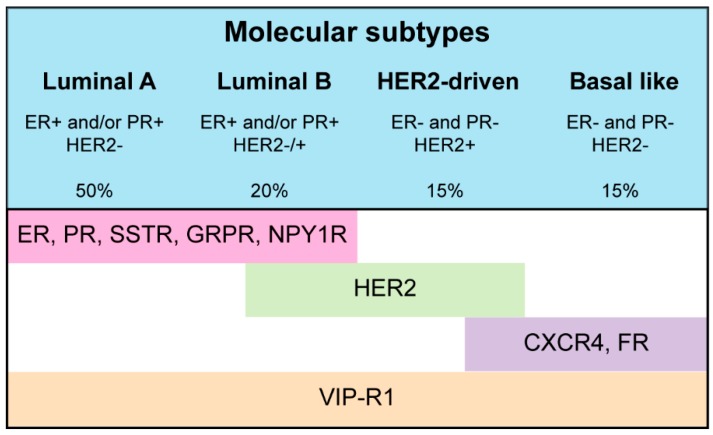
An overview of targets discussed in this review and the breast cancer subtype with highest expression thereof. ER: estrogen receptor, PR: progesterone receptor, SSTR: somatostatin receptor, GRPR: gastrin releasing peptide receptor, NPY1R: Neuropeptide Y receptor Y1, HER2: human epidermal factor receptor 2, CXCR4: C-X-C chemokine receptor type 4, FR: folate receptor, and VIP-R1: Vasoactive intestinal polypeptide receptor 1. Details on histological and molecular profiles are derived from [107].

One target does unfortunately not suit all BCs since this tumor type is very heterogeneous. Figure 4 and Figure 5 show an overview of the targets discussed in this review and the clinical setting in which radiotracers targeting these receptors can (potentially) be applied. Of the targets currently under investigation for receptor targeted nuclear imaging of BC, SSTR, GRPR, ER, PR, and NPY1R are best suited for ER-positive luminal A and luminal B BCs, since these targets are the only ones or the ones with the highest expression in these BC subtypes. Both ER- and PR-targeted radiotracers, but especially ER-targeted radiotracers, are currently studied in clinical trials. These radiotracers can be used for determining ER or PR expression of primary tumors and metastases as well as for evaluation of treatment response to ER-targeted therapy. However, the majority of ER-positive BCs acquire resistance against anti-estrogen treatment and in some cases this is due to loss of ER expression [105]. ER status of primary BCs and corresponding metastases may vary (over time) and so visualization of metastases of ER-positive primary tumors is not feasible in all cases [106]. ER- and PR-targeted imaging to determine receptor expression is less invasive than immunostaining on biopsy material as is done in clinical practice. Furthermore, ER- and PR-targeted nuclear imaging comprises visualization of the complete tumor lesion, while biopsy material is limited and not always representative for the (heterogeneous) tumor. However, determining hormone receptor expression with nuclear imaging would involve scanning patients several times with (different) radiotracers, which causes a significant radiation burden to the patient that has to be kept in mind. The application of radiotracers to determine hormone receptor status would therefore especially be beneficial for tumors that cannot be biopsied due to an inconvenient location. In cases where ER- and/or PR-targeted imaging cannot be applied, SSTR- and GRPR-mediated imaging, although not studied as widely as ER- and PR-mediated imaging, may be beneficial for imaging of ER-positive primary and metastatic BC lesions. GRPR-targeting is preferred because the receptor is expressed more frequently and at higher density [81]. Nevertheless, both SSTR- and GRPR-mediated imaging can be beneficial in ER-positive tumors that lose ER expression in the course of the disease. For this to be successful the relation between SSTR2, GRPR, and ER needs to be investigated to make sure that loss of ER expression does not influence GRPR expression. Furthermore, even though SSTR and GRPR expression is higher in ER-positive tumors, ER-negative tumors may also express the receptor and thus radiotracers targeting these receptors might also be applied in other patient groups. NPY1R expression was also associated with ER-positive BC’s. To date, NPY1R-targeted BC imaging has only been performed in a limited number of studies and more studies are needed to determine the added value of NPY1R in comparison to the above-mentioned targets for imaging of ER-positive BCs.

HER2-targeted BC imaging can be applied in HER2-positive BCs which account for approximately 15% of the BC population [105]. As previously mentioned, this approach can be used to determine HER2 expression and to select patients for therapy and to monitor response to treatment influencing HER2 expression. Similar to ER and PR radiotracers, determining HER2 expression with nuclear imaging can especially be beneficial in cases where biopsies cannot be obtained. Furthermore, as is the case for ER and PR, HER2 expression of primary tumors and metastases may change during the course of the disease [107].

CXCR4 and FR targeted nuclear imaging might be beneficial for basal-like tumors, the BC subtype with the worse prognosis [105]. CXCR4-mediated imaging in BC has not been successful up to date which might be caused by limited CXCR4 expression at the cell surface (necessary for radiotracer binding) or high CXCR4 expression in cancer stem cells of which only limited numbers are available in different BC subtypes [94]. Furthermore, high FR expression was also associated with basal-like tumors. Data on FR-targeting in BC is very limited, hampering discussion on the value of this radiotracer.

VIP-R1 is expressed on all BCs and thus radiotracers targeting VIP-R1 might be interesting for all BC subtypes. However, VIP-R1 is only expressed in early stage disease limiting the use of this tracer in advanced BC.

For the future, a noteworthy option might be the combination of radiotracers directed against different targets, the so called multi-target or “cocktail” approach, with the purpose of enhancing BC visualization. Reubi et al. [82] studied expression of SSTR, GRPR, VIPR-1, and NPY1R in human BC specimens and reported that 60% of the tumors expressed at least two of the targets. In this study, GRPR, NPY1R or both were expressed in almost all (93%) investigated BCs. Studies synthesizing and preclinically testing a hetero-bivalent dual target probe for GRPR and NPY1R were performed [108]. Next to these studies, other preclinical studies have investigated the multi-targeting approach [109,110,111], but to date this was not tested in a clinical setting. A disadvantage of this approach might be enhanced or more extensive uptake in healthy organs which naturally express these targets.

In addition to disease visualization, disease staging and evaluation of therapy response, another benefit of receptor targeted nuclear imaging is the use of radioligands for both imaging and therapy, following the so-called theranostic approach. Most of these radioligands can be labeled with imaging radionuclides (γ- or positron-emitters) as well as therapeutic radionuclides (β- or α-emitters), enabling the use of the same tracer for both imaging and therapy. This is especially interesting for treatment of advanced disease, since distant metastases are often not accessible for resection and most systemic agents are accompanied by severe side effects [3].

Furthermore, the use of dual labeled tracers that are labeled with both radionuclides and optical dyes are interesting for image-guided surgery. This can benefit surgical resection of tumors by offering preoperative imaging (SPECT or PET), intraoperative guidance (by making use of γ-probes detecting the radioactive signal to give an approximate tumor location), and fine guidance and tumor delineation (by detection of the optical signal), ultimately improving the success-rate of tumor resection.

## 8. Conclusions

Overall, receptor targeted nuclear imaging for BC imaging is promising and has the potential to improve BC care. There is not one appropriate target for all BCs, and thus a personalized approach should be applied. Depending on the BC subtype and the question of the physician, the appropriate target should be selected carefully (either by biopsy or imaging, depending on the availability of biopsy material). More studies are needed to directly compare the value of tracers targeting different receptors in specific patient groups—for example GRPR, SSTR, ER, PR, and NPY1R targeted imaging in ER-positive BCs and clinical studiescomparing their added value to currently available ^18^F-FDG PET/CT are needed to enhance their broad application in daily clinical routine.
